# Contextual interference in children with brain lesions: protocol of a pilot study investigating blocked vs. random practice order of an upper limb robotic exergame

**DOI:** 10.1186/s40814-020-00694-y

**Published:** 2020-10-15

**Authors:** Judith V. Graser, Caroline H. G. Bastiaenen, Urs Keller, Hubertus J. A. van Hedel

**Affiliations:** 1 Research Department, Swiss Children’s Rehab, University Children’s Hospital Zurich, Mühlebergstrasse 104, 8910 Affoltern am Albis, Switzerland; 2 Children’s Research Centre CRC, University Children’s Hospital, Zurich, Switzerland; 3 Research Group Function, Participation and Rehabilitation CAPHRI, Department of Epidemiology, Maastricht University, Maastricht, The Netherlands

**Keywords:** Paediatric neurorehabilitation, Motor learning, Contextual interference, Practice order

## Abstract

**Background:**

If adults practice several motor tasks together, random practice leads to better transfer and retention compared to blocked practice. Knowledge about this contextual interference (CI) effect could be valuable to improve neurorehabilitation of children. We present the protocol of a randomised controlled pilot study investigating the feasibility of blocked practice vs. random practice of robot-assisted upper limb reaching in children with brain lesions undergoing neurorehabilitation.

**Methods:**

Children with affected upper limb function due to congenital or acquired brain lesions undergoing neurorehabilitation will be recruited for a randomised controlled pilot study with a 3-week procedure. In the control week (1), two assessment blocks (robot-assisted reaching tasks, Melbourne assessment 2, subscale fluency), 2 days apart, take place. In the practice week (2), participants are randomly allocated to blocked practice or random practice and perform 480 reaching and backward movements in the horizontal and vertical plane using exergaming with an exoskeleton robot per day during three consecutive days. Assessments are performed before, directly after and 1 day after the practice sessions. In the follow-up week (3), participants perform the assessments 1 week after the final practice session. The primary outcome is the immediate transfer of the Melbourne Assessment 2, subscale fluency. Secondary outcomes are the immediate retention, 1-day and 1-week delayed transfer and retention and acquisition during the practice sessions. We will evaluate the feasibility of the inclusion criteria, the recruitment rate, the scheduling procedure, the randomisation procedure, the procedure for the participants, the handling of the robot, the handling of the amount of data, the choice of the outcome measures and the influence of other therapies. Furthermore, we will perform a power calculation using the data to estimate the sample size for the main trial.

**Discussion:**

The protocol of the pilot study is a first step towards a future main randomised controlled trial. This low risk pilot study might induce some benefits for the participants. However, we need to place its results into perspective, especially concerning the generalisability, as it remains questionable whether improving reaching constrained within a robotic device will ameliorate daily life reaching tasks.

**Trial registration:**

ClinicalTrials.gov Identifier: NCT02443857

## Background

The population of children undergoing neurorehabilitation is as heterogeneous as the causes for brain dysfunction. Differentiating between congenital and acquired brain lesions is common. Children with congenital brain lesions are diagnosed most frequently with cerebral palsy. The prevalence of cerebral palsy seems to decrease in Europe (from 1.90 to 1.77 per 1000 live births between 1980 and 2003); it is still the most common cause for severe motor impairments in children [1]. Acquired brain injuries in children are less common. In Norway, for example, the incidence for moderate traumatic brain injury amounts to 0.024 per 1000 children and for severe traumatic brain injury 0.025 per 1000 children [2]. For stroke, the worldwide incidence ranges between 0.013 and 0.13 per 1000 children [3]. Both, congenital and acquired brain lesions interfere with the normal development of the brain, which causes impairments in sensory-motor and cognitive functions and limitations in activities that may significantly impact quality of life [4]. Hence, these children should be referred to paediatric neurorehabilitation.

Nowadays, most neuro-rehabilitation programmes, including those for paediatric neurorehabilitation, are based on motor learning principles [5–7]. The general goal of motor learning is to attain relatively permanent changes in movement skills by practice and experience [8]. It has been suggested that the performance during practice (i.e. acquisition), retention (i.e. the preservation of the learned skill for a certain period) and transfer (i.e. when transferring the learned skill to another task) needs to be distinguished [9]. While retention is sustainability of performance after a practice phase, transfer reflects the effect of the practice on other, yet untrained, tasks [9]. Transfer is especially important since it allows generalising improved motor functions or capacities to daily life performance.

If several tasks have to be learned and are practiced within the same therapy session, they can interfere with each other, which could affect the outcome. In this so-called contextual interference effect [10], the practice order is an important factor that determines the strength of this effect. If one task is practiced several times before switching to the next task (i.e. practicing in a blocked order), contextual interference is low [10]. If different tasks are practiced in a random order, interference is high [10]. Contextual interference has mainly been investigated in healthy adults where it was shown that high contextual interference leads to worse performance in acquisition but better performance in retention and transfer, while low contextual interference leads to contrary results [11, 12]. Two main hypotheses explaining these findings have been discussed in the literature. On the one hand, the elaborative-processing hypothesis states that compared to blocked practice, the learning process during random practice is based on a more-profound elaboration of the tasks due to the comparisons between and within the trials [13]. This could lead to a more comprehensive memory trace, which is easier to retrieve [14]. The forgetting-reconstruction hypothesis, on the other hand, is based on a strengthened memory consolidation occurring during random practice [15]. The underlying explanation is that during random order practice, the learner switches between different tasks all the time and forgets the established action plan of the prior task when a subsequent new task is performed [14]. It seems comprehensible that (a) a deeper elaborated and more robust memory representation could result in better retention and transfer and (b) acquiring a motor skill under blocked practice might be easier because of less disturbance, leading to a better momentary performance at acquisition compared to random practice.

It has been stated that the contextual interference effect is larger when the tasks involve different motor programmes [11, 16]. A motor programme can be understood as a memory for a movement class rather than for an action or a movement solely [17, 18]. Actions that have the same invariant aspects in common (e.g. spatial and topographical characteristics of the action [19], relative force and timing and sequences involved in the action [11]) are controlled by the same motor programme [11, 16]. Therefore, applying variations of the invariant features would increase the contextual interference effect. However, as contextual interference has mainly been investigated in healthy adults, evidence is lacking for children with congenital or acquired brain lesions [20].

Adhering to the recommendation of providing intensive and repetitive training to restore motor function in neurorehabilitation [21] is challenging, especially when working with children. To keep them engaged during highly repetitive therapy sessions, practice has to be variable. To induce variability, several tasks or variations of a task are practiced usually within one therapy session. Since we aim to provide efficient therapies to obtain optimal outcomes, we consider it relevant to improve our understanding of the influence of contextual interference on the therapeutic outcome of paediatric neurorehabilitative interventions.

In recent years, rehabilitation technologies have been applied increasingly also in paediatric neurorehabilitation [22]. Main advantages are the standardisation of training protocols and assessments, high number of repetitions, and above all, additional motivation due to exergames [22]. While the definition of ‘exergame’ is debated, in this study, we use the common definition ‘videogames that require physical activity in order to play’ p. 10 [23]. To our knowledge, two studies used new technologies to investigate the influence of the practice order. The first study investigated motor learning in children with developmental coordination disorder and typically developing children. The children practiced with the Nintendo® Wii Fit video game during 10 20-min sessions in a variable group (a self-selected choice out of 10 games) and a repetitive group (the same game throughout the whole session) [24]. In the second study, children with cerebral palsy and typically developing children practiced a computer-maze in random order (30 trials, 5 different mazes in random order) and in constant order (30 trials of 1 maze) [25]. While both practice groups improved their game performance similarly in the first study [24], the random order group showed a reduction in movement time needed to complete the maze tasks in retention and transfer in the second study [25]. However, as both studies did not include a true blocked group, we designed a study protocol to investigate blocked vs. random order. As there is not much knowledge in the field of contextual interference with robotic exergames in paediatric neurorehabilitation, we planned a pilot study to evaluate the feasibility of a future main study. The objective of this paper was to present *the protocol* of this pilot study evaluating the feasibility of a randomised controlled, single-blinded study about contextual interference in robot-assisted upper limb training in children with congenital or acquired brain lesion and affected upper limb function. To assess the feasibility, we aimed to address the following questions:

1. Are the chosen inclusion criteria specific enough to result in a sample of participants, which is suitable for this pilot study?
2. Is the recruitment rate feasible?
3. Is the scheduling procedure feasible?
4. Is the randomisation procedure feasible?
5. Is the whole procedure feasible for the participants?
6. Is the handling of the robot feasible?
7. Is the handling of the large amount of data feasible?
8. Are the outcome measures responsive and sensitive enough within this setting?
9. Is there a confounding influence of parallel therapies within the rehabilitation setting?
10. Is it feasible to conduct the main trial with respect to the needed sample size calculated from the data obtained for the primary motor learning outcome?

## Methods

### Design

This pilot study protocol describes a randomised controlled single-blinded two-arm intervention study comparing several outcome time points between two groups of children with neuro-motor upper limb impairment who will practice two variations of reaching movement tasks with robot-assisted exergames (one group in blocked, the other in random order) with a predefined primary outcome and a follow-up period of 1 week. We will include a follow-up period of 1 week to match the rather short intervention period; 1 week will also be feasible to plan within a rehabilitation stay.

### Setting

The study will take place at the Swiss Children’s Rehab of the University Children’s Hospital Zurich in Affoltern am Albis, Switzerland. All measurements and practice sessions will be performed during a multidisciplinary in-patient rehabilitation stay.

### Ethical considerations and reporting

This study has been approved as part of the ChARMin project (sub-project 5: Motor learning) by the Ethics Committee of the Canton Zurich (BASEC-Nr. PB_2016-02450) and the Swiss Agency for Therapeutic Products (Swissmedic reference number: 2015-MD-0009).

According to the Ethics Committee’s guidelines, all the participants will give their verbal consent, children of 15 years and older and all the legal representatives will provide informed written consent.

**Fig. 1:**
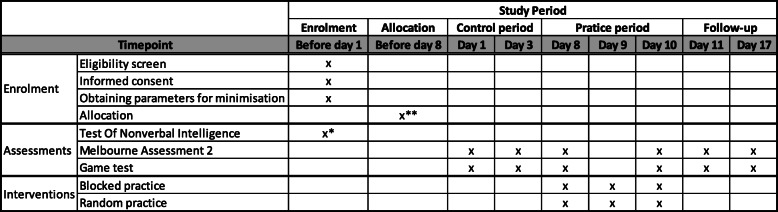
Standard Protocol Items Recommendation for Interventional Trials (SPIRIT) figure. Actions and appointments throughout the whole study. *The test of nonverbal intelligence is conducted before the first practice session (outcome needed for randomisation by minimisation). **Allocation is done when all the parameters for minimisation are obtained; at latest on the day before the first practice session

This study protocol was established according to the guide on reporting protocols of pilot and feasibility trials [26], the Consolidated Standards of Reporting Trials (CONSORT) statement extension for randomised pilot and feasibility trials [27] and the Standard Protocol Items: Recommendations for Interventional Trials (SPIRIT) [28]. See Additional file 1 for the SPIRIT checklist and Fig. 1 for the SPIRIT Figure.

### Participants

The majority of children admitted to our rehabilitation centre has a congenital brain lesion (cerebral palsy) but we treat also children with acquired brain lesions (after stroke or traumatic brain injury), spinal cord injury, genetic syndromes, etc. The reasons for admitting a patient vary. Most children with cerebral palsy come to the centre for a shorter period (4 to 6 weeks) of intensive therapy or after orthopaedic surgery, particularly of the lower limbs, with consecutive rehabilitation. Patients who experienced an acquired brain lesion, such as a stroke or traumatic injury, are admitted as soon as they are stable and can leave the intensive care unit. Depending on the recovery, some of these patients might also be re-admitted for rehabilitation at a later stage. Currently, the average length of stay is around 40 days but varies from a couple of weeks to more than a year (for children with acquired brain lesions). According to their rehabilitation goals, the children undergo physiotherapy, occupational therapy, sports therapy, speech and language therapy, neuropsychology, hippotherapy, and robot-assisted therapy. For this pilot study, we will recruit a sample representing patients with congenital or acquired brain lesions, reflecting the majority of our inpatient population.

#### Inclusion and exclusion criteria

Included will be children with either congenital or acquired brain lesions. Those with an acquired brain lesion should be in the subacute, i.e. more than 3 months, or chronic stage. Further inclusion criteria will be uni- or bilaterally affected upper limb function with spasticity, dyskinesia or mixed conditions. Additional inclusion criteria will be age 5 to 18 years, the ability to sit upright for approximately 60 min without lateral trunk support and a Manual Ability Classification System (MACS) level between I and IV (MACS level I: handles objects easily and successfully; level II: handles most objects but with somewhat reduced quality and/or speed of achievement; level III: handles objects with difficulty, needs help to prepare and/or modify activities; level IV: handles a limited selection of easily managed objects in adapted situations [29]).

Moreover, the children will need to be able to understand and follow test instructions, be compliant for the whole study procedure, can communicate pain or discomfort and see a computer screen at approximately 1 m in front of him/her. Excluded will be children with upper limb surgery or Botox injections during the past 6 months, and upper limb skin lesions.

We decided not to include outpatients since we learned from prior experiences that it is difficult for parents to organise their children’s attendance for participating in such an extensive study procedure.

### Target sample size

Since the recruitment rate will be one of our feasibility criteria, we will recruit for 1 year and calculate the recruitment rate, taking into account the number of eligible and recruited participants, and complete datasets. Yet, the CONSORT 2010 statement extension to randomised pilot and feasibility trials recommends to give some rationale for the target sample size [27]. Based on our center’s numbers from past years and taking into account the inclusion criteria, we anticipate to recruit 20 participants (10 per group).

### Recruitment

We will inform the children who are admitted to our centre matching the inclusion criteria and their parents/legal representatives about the study. If they provide their written informed consent, the children will be included and the appointments scheduled.

### Group allocation

Participants will be allocated to a blocked or random practice group using randomisation by minimisation. This method enables balancing several prognostic factors in small samples [30]. We will impute parameters that potentially influence motor learning. To reduce the risk of selection bias, these factors should be balanced between the groups, even in the case of uneven distribution.

We will use the following minimisation parameters: age (preschool age: 5–6 years, primary school age: 7–12 years, secondary school age and older: 13–18 years); gender (female, male); diagnose (congenital, acquired); manual ability (MACS level I, II, III or IV); and cognitive ability according to the Test of Nonverbal Intelligence–Fourth edition (TONI-4), which evaluates abstract reasoning and problem solving (Index Score < 70: very poor, 70–79: poor, 80–89: below average, 90–110: average, 111–120: above average, 121–130: superior and > 130: very superior) [31]. The minimisation parameters are equally weighted. A study nurse will receive the minimisation parameters of each new participant by one of two researchers involved in recruitment. She will enter the parameters in a custom-written Matlab programme, which will allocate the participant to either the blocked or random order group. The study nurse will be unaware of this definition and the purpose of the study. The study nurse will enter the allocation in a file, which will allow the researcher to schedule the appointments and perform the practice and measurement sessions. The study nurse will not be involved otherwise in this trial. In case the study nurse is unavailable, we will assign another independent person who will perform this procedure.

### Equipment, intervention, outcome measures and study procedure

#### Robot

**Fig. 2:**
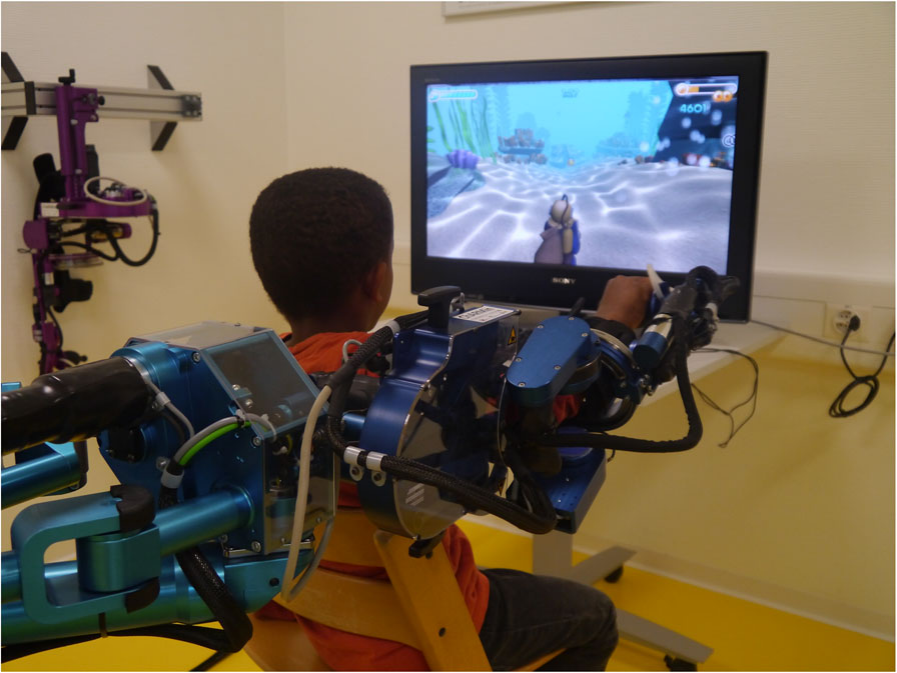
The ChARMin robot. The ChARMin exoskeleton: a young boy practicing with ChARMin’s small distal module

We selected the ChARMin (Children’s Arm Rehabilitation Mechatronic Interface) device that was developed in a collaboration between the Sensory-Motor Systems Lab (Federal Institute of Technology Zurich, Switzerland) and the Swiss Children's Rehab [32]. ChARMin (Fig. 2) is an actuated exoskeleton robot, which can actively support joint movements of the shoulder, elbow, forearm and wrist for the left or the right arm. To support the patient’s movements, the motors compensate the weight and friction of the robot arm and can provide guidance force. Therapists can adjust the guidance from a non-supporting mode, where the robot transparently follows the patient’s movement, to a fully supported mode. This allows adapting the physical support of ChARMin to each patient during therapies. However, for the planned pilot study, participants will not receive physical guidance or support of the device; the motors will only compensate for the weight and friction of the robot.

ChARMin includes a small and large distal module to fit the anthropometrics from about 5-year-old children to adolescents. Interfaced with different exergames, it provides and facilitates playful training of arm and hand functions, especially for children with more severe upper limb impairments. During the execution of the exergames, different sensors and game parameters are recorded and processed to extract the assessment measures (e.g. number of velocity peaks, precision on the target or reaction time).

#### Intervention

We selected an exergame for the pilot study that is based on the Quality of Movement assessment provided by ChARMin [33]. The participants will need to perform goal-directed reaching movements. The participants will have to steer an avatar, which represents the position of the participant’s hand, on the screen by moving the upper extremity with the attached exoskeleton towards one of eight targets appearing in random order radially around the centre. The children will be instructed to reach the targets by the most direct path and as fast as possible. The avatar will need to remain on the target for 2 s. After that, the target will disappear. Then, the centre object will reappear, and the avatar will have to be moved back to the centre object to remain there for another 2 s before the next target will appear. If the participant will not reach the target within 10 s, it will disappear, and the centre object will reappear.

Since interference levels must be appropriate to produce a contrast between random and blocked schedules [34], we developed two versions of the exergame; one can be played in the transversal plane, the other in the frontal plane (i.e. the two versions of the exergames have different spatial characteristics, which should require different motor programmes) [11].

**Fig. 3:**
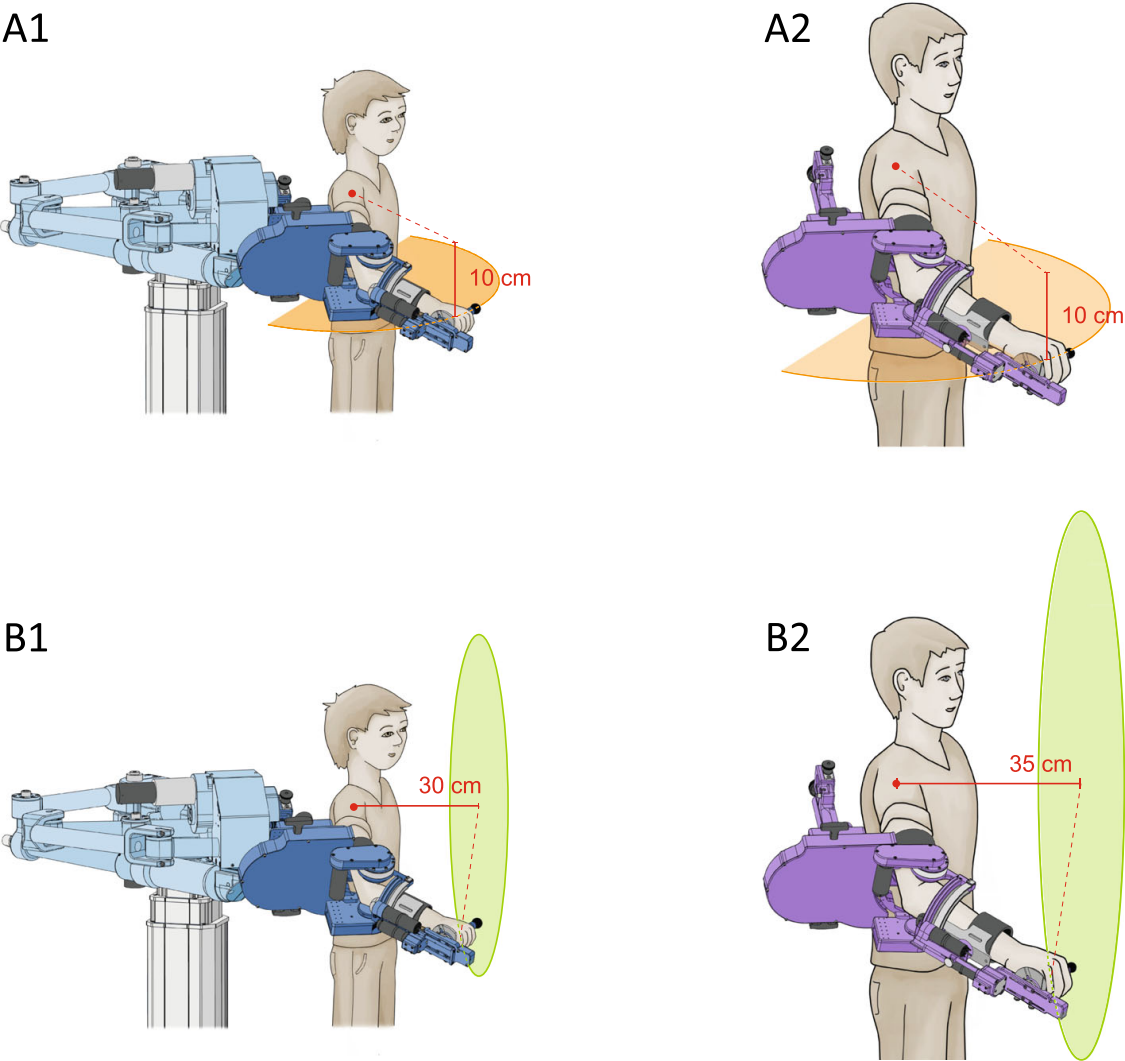
Restrictions of movement inside a haptic wall on the horizontal (A) and the vertical plane (B). The movement area during the horizontal plane exergame with the small distal module (A1) and the large distal module (A2). The haptic wall is located 10 cm below the shoulder joint. The movement area on the vertical plane is located 30 cm in front of the shoulder joint for the small distal module (B1) and 35 cm in front of the shoulder joint for the large distal module (B2)

Choosing different varying characteristics would be an option, and has already been evaluated [35]. Including variations based on different motor programmes has been suggested to create a more difficult learning situation [11]. As we did not want to increase the learning difficulty too much for the participants, we decided to vary the tasks only in one parameter. In the transversal plane version, the exergame will be displayed as a horizontal plane on the screen. The participant will move inside a haptic wall, i.e. the movement will be restricted mechanically to a horizontal plane, which will be located 10 cm below the shoulder joint for the small and the large distal module (Fig. 3(A1 and A2)). Mainly horizontal shoulder adduction and abduction, and elbow flexion and extension will be required to play the horizontal version of the exergame. In the frontal plane version, the exergame will be displayed vertically on the screen. Here, the participant will move inside a haptic wall located 30 cm in front of the shoulder joint for the small distal module and 35 cm in front of the shoulder joint for the large distal module (Fig. 3(B1 and B2)). Shoulder flexion and extension, (horizontal) abduction and adduction, shoulder internal- and external rotation and some elbow flexion and extension will be required to play the vertical version of the exergame.

**Fig. 4:**
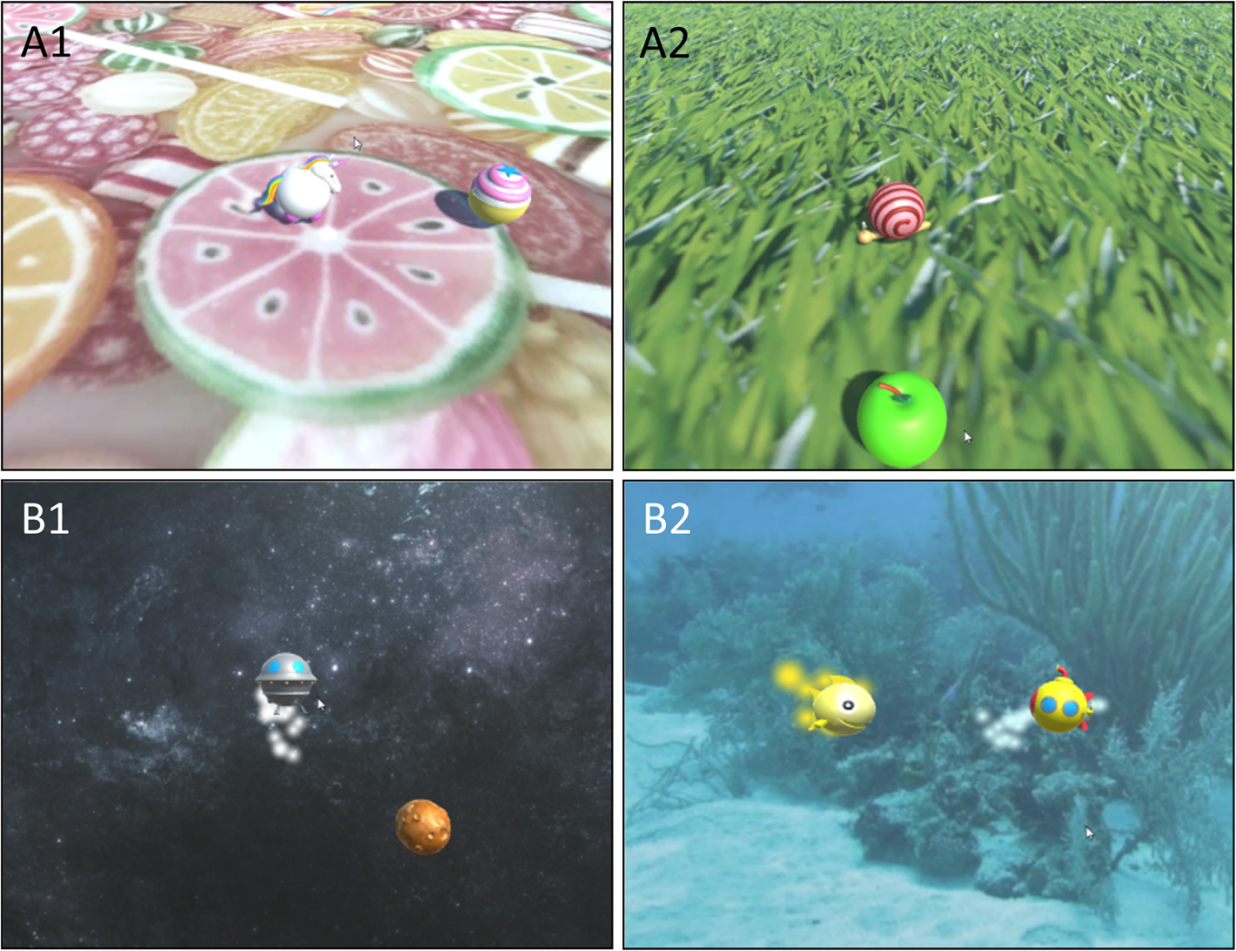
ChARMin exergames. Upper row: transversal version of the ChARMin exergames (A1: avatar unicorn, target cupcake; A2: avatar snail, target apple). Lower row: frontal version of the ChARMin exergames (B1: avatar UFO, target planet; B2: avatar submarine, target fish, in this picture the gold target is displayed)

To motivate the participants to perform a large number of repetitions actively, we developed different avatar-target-exergame-scenarios (Fig. 4). Prior to the exergame, the participant will select his/her own avatar to improve compliance. For the transversal plane exergame version, the avatar will either be a unicorn who will be eating cupcakes (targets) or a snail eating apples. For the frontal plane exergame, the avatar will be an UFO landing on planets or a submarine collecting fish. As previously mentioned, we will instruct the participants to ‘move the unicorn/snail/UFO/submarine to the cupcake/apple/planet/fish as direct and fast as possible’.

Reaching a target will be rewarded with a score displayed in the middle of the screen. The score will be calculated using the time remaining to reach the target (0 to 10 s), and the distance to path ratio (1 = direct movement to the target):

$$ \mathrm{Score}=\mathrm{time}\ \mathrm{remaining}+\left(11-\frac{\mathrm{path}}{\mathrm{distance}}\right) $$

Therefore, the maximum score for each target will be 20. A randomly chosen target out of the eight targets per trial will be a golden target with a value worth five times as much to increase the participant’s motivation. The current score of the ongoing trial will be displayed in the left upper corner of the screen. The total score will be displayed after each trial and will not exceed 240 points.

The blocked group will play 15 trials of one version (i.e. in either the horizontal or vertical plane) followed by 15 trials of the other version. The order of the two blocks will be randomised and the same for all the practice sessions for the same participant. The random group will play the 30 trials of the two versions in a pseudo-random order. The only restriction will be that the same version cannot be repeated more than twice in a row.

To generate observable changes due to motor learning, a large number of repetitions (i.e. hundreds of daily repetitions for upper extremity movements) will be required [36, 37]. Results from a recent study proposed changes in performance scores during three sessions, each lasting 20–25 min of robot-assisted arm training, and showed that movements were repeated more than 3000 times [38]. Yet, it is challenging to define the number of repetitions sufficient to induce motor learning, while maintaining an acceptable length of sessions to keep the burden for the participants at a minimum and their motivation at a maximal level. In our study, participants will perform 16 reaching movements within one trial of exergames. Over the three sessions (30 trials per session), this would lead to 1440 repetitions of the reaching movement. We consider that this number will induce change due to motor learning. On the other hand, we expect a maximum session duration of 120 min, which seems acceptable.

When switching between the two exergame versions, the therapist will not need to adjust the hardware settings, but she will need to choose the appropriate version of the exergame on the interface. In order to have the same conditions for all participants, we will renounce using the support modalities of the robot. While children with unilaterally affected upper limb function will practice and perform all the assessments with their more affected side, children with bilaterally affected upper limb function will use the arm which they subjectively use more in daily life.

#### Outcome measures

##### Outcomes of the main study

***Primary outcome: immediate transfer of the practiced skill*** We chose the Melbourne Assessment 2 subscale fluency (MA2_fluency_) as the primary outcome measure for the immediate transfer. The MA2 consists of the four subscales movement range, accuracy, dexterity and fluency [39] and is an adequate measurement for research and clinical use in children with cerebral palsy [40]. The intraclass correlation coefficient of the test-retest reliability of the MA2_fluency_ is 0.96 (95% confidence interval 0.90–0.99), the minimal detectable change is 2 points and the minimal clinically important difference is at least 3 points [40]. Concerning the concurrent validity, Pearson’s correlation coefficients between the MA2_fluency_ and other tests amounted to 0.67 (Bruininks-Oseretsky Test of Motor Proficiency, Second Edition, subtest 3, manual dexterity), 0.76 (Box and Blocks Test) and 0.40 (Paediatric Motor Activity Log–Revised, quality of movement) [40]. The responsiveness of the MA2_fluency_ is high, which is indicated by significant change scores and a high standardised response mean value of 1.84 [40].

From a clinical point of view and in line with literature (e.g. [9]), we consider the transfer to be an important aspect of learning. We hypothesise that MA2_fluency_ will improve when practicing the reaching movements. During the exergames, the participant will be instructed to reach the target by the most direct path, i.e. as fast as possible. This is in line with the MA2_fluency_ scoring criteria, which include jerkiness, tremor and/or reduced speed of movement [41].

Since we considered the MA2_fluency_ to be more sensitive to change instantly after the practice sessions due to the relatively short practice period and to avoid a potential loss to follow-up at later time points, we chose the immediate transfer as the time point of interest for the primary outcome.

One of three trained occupational therapists will score the MA2_fluency_ by analysing the video. The rater will be blinded for the participant’s group allocation, the time point of assessment and the randomisation code. The sum scores will be calculated and used for further analysis.

***Secondary outcomes*** *Delayed transfers*: The delayed transfers (1 day and 1 week after the last practice session) will be determined by the sum score of the MA2_fluency_ at these time points.

*Immediate and delayed retentions*: To evaluate whether the participants can retain what they have learned, the so-called exergame test will be performed. It consists of one block of three trials of the horizontal version of the exergame and one block of three trials of the vertical version of the exergame. Although the order of the versions will be randomised between the participants, it will remain the same within each participant throughout the study.

With these exergame tests, we anticipated measuring the construct ‘movement fluency’. There is no generally accepted way of quantifying fluency. Several studies have used the absolute number of velocity peaks of a single movement [42, 43]. Yet, as we also wanted to take into account the length of the movement path, we will calculate the parameter ‘number of peaks’, which is the number of velocity peaks (i.e. when the difference between a local speed minimum and a local speed maximum exceeds a value of 2.5% of the measured maximal speed) normalised to the covered distance of the movement path (nP_norm_). We will calculate nP_norm_ for each movement (i.e. one value from the centre to the target and one for the return to the centre starting point) that will lead to 16 data points per trial. The parameter nP_norm_ has shown reliable results during the Quality of Movement assessment [33], on which the exergames are based. In a comparable group of 23 participants, the parameter nP_norm_ showed no systematic error between two measurements within 3 to 7 days, an intraclass correlation coefficient of 0.94, a percentage standard error of measurement (divided by the grand mean) of 6.4% and a percentage smallest real difference (divided by the grand mean) of 17.7% (unpublished data).

*Acquisition*: To observe motor learning during the practice sessions, nP_norm_ will be the parameter of interest too. It will also be recorded 16 times per trial, which will lead to 480 data points per practice session.

##### Feasibility outcomes

In line with recommendations on reporting protocols of pilot and feasibility trials [26], we will apply the following ten specific feasibility criteria:

1. Are the chosen inclusion criteria specific enough to result in a sample of participants, which is suitable for this pilot study (e.g. are they able to play the exergame)?
2. Is the recruitment rate feasible? Since the main randomised controlled trial would require a large number of participants, it is relevant to know the number of participants that will be recruited during a certain period.
  The number of children enquired for the study, number of participants recruited, number of complete datasets within 1 year will be recorded and used to calculate recruitment rates.
3. Is the scheduling procedure feasible? The appointments will be planned for 3 weeks for each participant. The person conducting the exergame tests and the MA2_fluency_ always will need to be the same. The same will apply for the person instructing the child during the practice sessions. Absences of personnel (e.g. due to illness, holidays or part-time employment) and the children (e.g. due to illness or short-term planned medical appointments), as well as the availability of the robot and material for the MA2_fluency_ (i.e. whether they are not used for therapy at the time points of the measurements), will need to be considered. Information about whether it is possible to schedule rooms, persons involved and the robot and table for performing assessments will be noted.
4. Is the randomisation procedure feasible? Experiences of the persons involved will be collected and evaluated to answer this question.
5. Is the whole procedure feasible for the participants? The rather time-consuming procedure and the high number of repetitions might be difficult for at least some of the participants because of fatigue or lack of motivation to play the exergames so many times. Both reasons influence motor learning negatively. Therefore, it is important to determine whether the participants included in this study are able to comply the whole study procedure. Information about aborted or shortened sessions or other incidents and their reasons (e.g. pain or discomfort, fatigue) will be taken from the lab journal.
6. Is the handling of the robot feasible? The ChARMin robot has been in use in the clinical and research area since 2015. Since it is a newly developed tool, it is important to evaluate its handling. Any technical issues will be noted and then retrieved from the lab journal.
7. Is the handling of the large amount of data feasible? Information from the assessors and the analyst on the primary outcome will be used.
8. Are the outcome measures responsive and sensitive enough within this setting? Responsiveness analysis of the assessment data will be performed.
9. Is there a confounding influence of parallel therapies within the rehabilitation setting? As the study will be conducted during the inpatient rehabilitation stay, it is important to estimate whether parallel-applied therapies could confound the results. Since an additional control group following usual care would require a larger sample, we chose a design in which each participant acts as its own control and, therefore, we included control measurements during the first week of the study procedure.
  The changes in the outcomes between week 1 (control week) and week 2 (practice week) will be compared. The kind and number of additional therapies taking place during the different study periods will be recorded to increase the quality of reporting [44].
10. Is it feasible to conduct the main trial with respect to the needed sample size calculated from the data obtained for the primary motor learning outcome? With the data of the primary outcome measure, we will calculate the required sample size for the main trial.

#### Study procedures

**Fig. 5:**
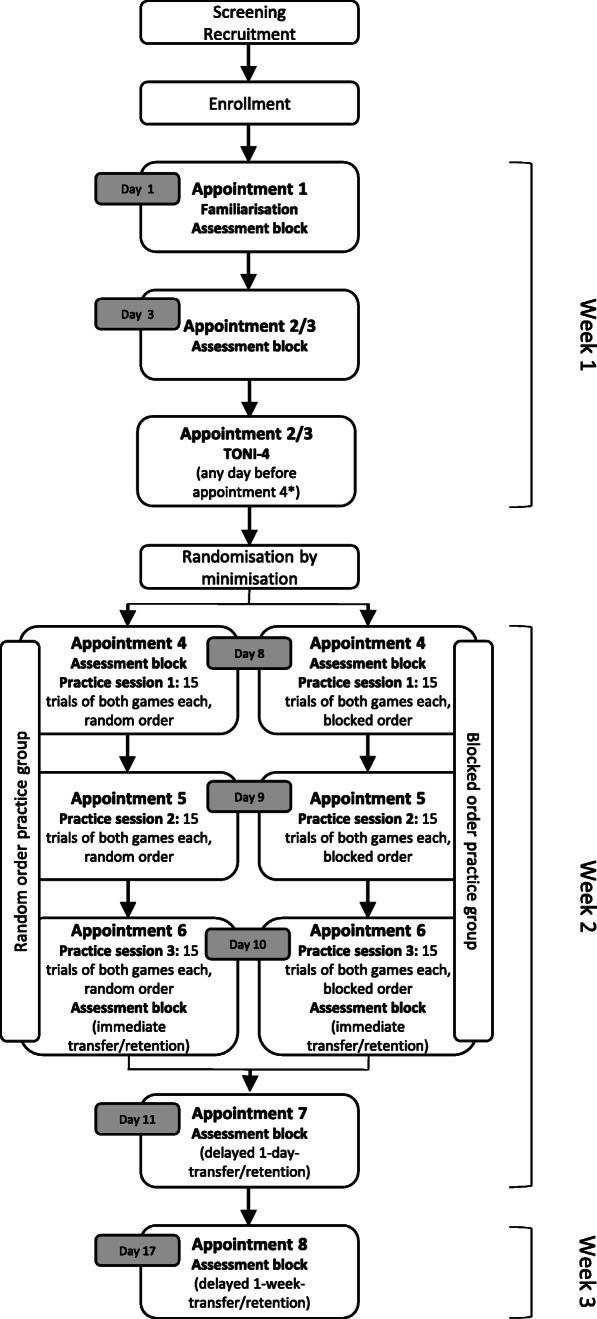
Study procedure. The study duration for each participant is 3 weeks. Week 1 contains the familiarisation of the two versions of the exergame, two assessment blocks (Melbourne Assessment 2 and exergame tests) and the Test of Nonverbal Intelligence, Fourth edition (TONI-4). The TONI-4 will be planned before the first practice session day (i.e. on appointment 2 or 3). Week 2 contains three practice sessions with an assessment block proceeding the first practice session and another assessment block after the last practice session to evaluate immediate transfer and retention. One day after the last practice session, the assessment block is repeated to assess the delayed one-day transfer and retention). During week 3, the last assessment block is scheduled one week after the last practice session to assess the delayed 1-week transfer and retention. *TONI*-*4* Test of Nonverbal Intelligence, Fourth edition

The study procedure is shown in Fig. 1 (SPIRIT Figure) and Fig. 5.

##### Week 1

During the first appointment, the setup of the robot and the adjustments according to the child’s anthropometrics will be performed. This takes approximately 15 min. Each participant will then become familiarised with the two versions of the exergame. First, the transversal version ‘Unicorn’ will be played with manual guidance and verbal support from the therapist. Then, the transversal version ‘Snail’ will be played with only verbal guidance of the therapist. The same procedure will be followed for the exergame that is played in the frontal plane (i.e. ‘Submarine’ will be played with manual guidance and verbal support followed by ‘UFO’, which will be played with only verbal support). Subsequently, an assessment block consisting of the MA2_fluency_ [45] and an exergame test will be performed.

During week 1, also all the parameters for randomisation by minimisation will be obtained from the participant’s medical history (e.g. age, gender, diagnosis), from the attending occupational therapist (MACS level), and trained neuropsychologist (TONI-4). Participants will be characterised by age, gender, weight, height, diagnosis (including information about the affected side and specific features such as spasticity, dyskinesia or mixed condition) and the MACS level. After obtaining the minimisation parameters, participants will be allocated to their group.

##### Week 2

On each of three consecutive days, all participants will practice 30 trials (15 trials of the horizontal and 15 of the vertical version of the exergame) in either a blocked or random order (according to their group allocation). Right before the first and after the last practice session, an assessment block will take place to assess the acquisition and immediate transfer and retention. One day after the last practice session, the assessment block will be repeated (1 day delayed transfer and retention).

##### Week 3

One week after the last training session, the assessment block will be repeated (1 week delayed transfer and retention).

The first session will include robot set-up and familiarisation and will be scheduled for approximately 75 min. Assessment blocks will take approximately 35 min and practice sessions 60 to 90 min. As the duration of an exergame trial depends on the child’s performance, it will vary largely and will affect the overall duration of the practice session. Once the robot settings have been adjusted to the participants’ anthropometrics, no additional set up time will be required. We defined an upper time limit of 120 min per session, which could be reached during practice sessions 1 and 3 that will also include a previous or subsequent assessment block. Sessions will be aborted after 120 min.

##### Standardisation and blinding

All the appointments will be scheduled during the same half of the day throughout the 3 weeks to reduce the influence of daytime on the outcome (e.g. fatigue increasing throughout the day, the severity of spasticity changing over the day, etc.). A trained physiotherapist or human movement scientist will conduct the practice sessions and the assessments following standardised instructions. The same person will conduct at least all assessment blocks with a participant and, whenever possible, also the practice sessions. In each session, an assistant will note irregularities and support the therapist.

While the assessor of the primary outcome (video analysis of the MA2_fluency_) will be blinded to the participants’ allocation and the time-point of the assessment, the participants will be blinded to the detailed aims of the study. Both will be informed that the intervention will investigate motor learning with new exergames but not about our interest in investigating the effects of different practice orders.

### Data analysis

**Fig. 6:**
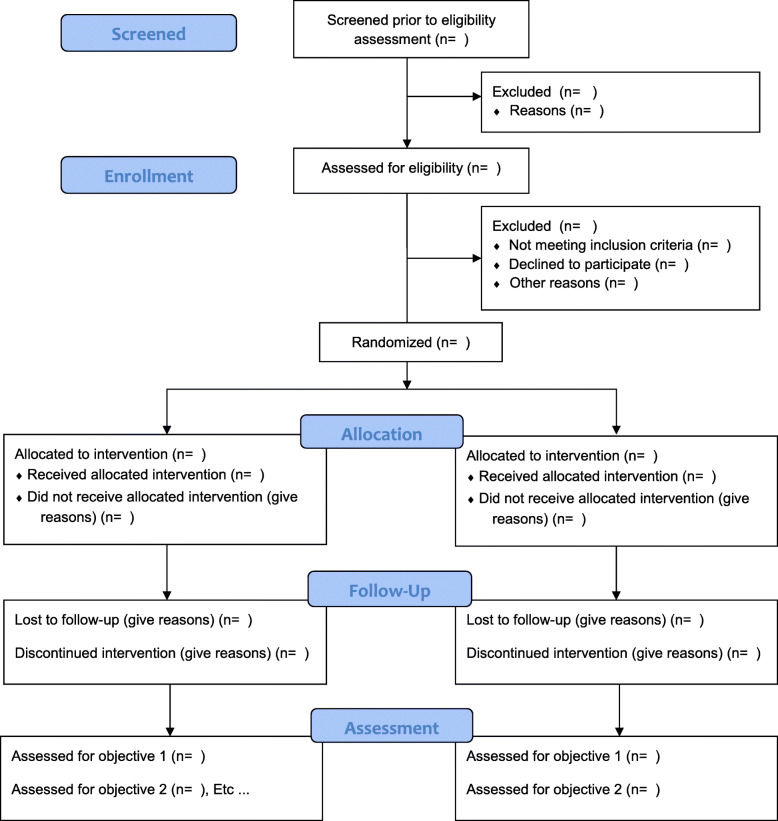
The Consolidated Standards of Reporting Trials (CONSORT) extension to randomised pilot and feasibility trials flow diagram [27]

We will present the numbers of the recruitment and measurement procedure in the CONSORT extension to randomised pilot and feasibility trials flow diagram (Fig. 6). We will report the participant characteristics and the baseline descriptives for each participant.

ChARMin data will be processed by a custom-made Matlab algorithm (The MathWorks Inc.). Statistical analysis will be performed with IBM SPSS Statistics 24.

In case of incomplete datasets, loss to follow-up or if a participant would be treated in the other treatment group than the one he or she was allocated to, we plan an intention-to-treat analysis. In case of missing data (e.g. a participant is not able to attend a session), we plan to perform a multiple imputation by chained equation.

#### Analysis of feasibility

The ten areas (see outcome measures section) will be evaluated with the information obtained during the study. Only questions 8, 9 and 10 will require statistical analyses to be answered:

8) Responsiveness analysis of the outcome measures for this specific intervention: the data (MA2_fluency_ and exergame test data) of the measurement time point immediately before the first and right after the last practice session will be compared. For internal responsiveness, the standardised response mean will be calculated. For the external responsiveness, a Pearson correlation coefficient will be calculated between the change-scores (i.e. between the two measurement time points) of the MA2_fluency_ and the ChARMin parameter nP_norm_.
9) Effect due to the parallel therapies within the rehabilitation setting: We will include data of all participants to test for a significant change in exergame and MA2fluency scores between day 1 and day 3 in week 1 (i.e. without specific intervention, see Fig. 4) using a paired *T* test or Wilcoxon signed-rank test if a non-parametric analysis will be required. Assuming that the participants will have similar therapy schedules during week 1 and 2, and no change is measured during week 1, a change during week 2 could be attributed to the robotic training. However, if outcomes will improve significantly in week 1, changes observed during week 2 will need to be interpreted with caution, as they cannot be attributed to the study’s intervention alone.
10) The sample size estimation for the main trial will be calculated according to the formula

$$ n=\frac{{\left({Z}_{\alpha }+{Z}_{\beta}\right)}^2\times 2{\sigma}^2}{{\left({\mu}_1-{\mu}_2\right)}^2}\kern0.75em $$[46].

Where *n*= sample size, *Z*_*α*_= standard normal *z* value for a significance level *α* = 0.05, which is 1.96, *Z*_*β*_ = standard normal *z* value for the power of 80%, which is 0.84. The pooled standard deviation of the pre-and post-intervention differences (both groups) is indicated by *σ*, *μ*_1_ is the mean pre-post-intervention difference of the intervention group 1, *μ*_2_ is the mean pre-post-intervention difference of the intervention group 2.

#### Analysis of motor learning outcomes of the main trial

As recommended by the CONSORT extension to pilot and feasibility studies, we will report 95% confidence interval estimates of the motor learning outcomes [27], as the study will be most likely underpowered. Hypothesis testing will only be conducted if the recruited sample size is large enough to warrant sufficient statistical power.

##### Primary outcome

***Immediate transfer*** The relative difference $\left(\frac{MA{2}_4- MA{2}_3}{MA{2}_3}\right)$ of the MA2_fluency_ sum score between the time point right before the first practice session (MA2_3_) and the time point immediately after the last practice session (MA2_4_) will be compared between the groups. A *T* test (or Mann-Whitney *U* test, if data are not normally distributed) will be performed.

##### Secondary outcomes

For all secondary outcomes, a multifactorial repeated measures analysis of variance (ANOVA) with ‘practice group’ and ‘time point’ as factors will be conducted over all the time points. A post-hoc analysis (Bonferroni correction for multiple comparisons) will be used to test for significant changes between the appropriate time points.

***Delayed transfers*** The MA2_fluency_ sum scores will be compared between the time point right before the first practice session, 1 day after the last session (1-day-delayed transfer), and 1 week after the last session (1-week-delayed transfer).

***Immediate retention, delayed retentions, acquisition*** For the several retention time points and the acquisition, the parameter nP_norm_ (obtained during the exergame tests and the practice sessions, respectively) will be evaluated. Comparisons will be made between the data obtained during the exergame tests right before the first practice session with the data from immediately after the last practice session (immediate retention), 1 day after the last practice session (1-day retention), and 1 week after the last practice session (1-week retention). For the acquisition, all the data points obtained during the practice trials will be compared.

##### Assumptions

Generally, statistical testing is only performed in case of sufficient statistical power. For a *T* test, the normal distribution of the data will be required, which will be tested with a Shapiro-Wilk test. For an ANOVA, the homoscedasticity of values of the groups and normal distribution of the population of the residues will be tested with the Levene test and the Shapiro-Wilk test, respectively.

## Discussion

We aimed to present the study protocol of a randomised controlled single-blinded pilot trial to explore the feasibility of testing the contextual interference effect while practicing robot-assisted upper limb tasks with exergames in a representative sample of children with congenital or acquired brain lesions.

### Putting the pilot study in the wider context

It is common that different motor tasks are practiced within the same therapy session in paediatric neurorehabilitation. Human movement is variable (e.g. [19]) and variability generally seems to be beneficial for learning of new motor skills [47]. Despite this, high-level evidence about the contextual interference effect in children in general and particularly in children with brain lesions is missing [20]. If children with congenital or acquired brain lesions would respond similarly as healthy adults to the contextual interference effect, random practice would be more beneficial compared to blocked practice at retention and transfer. On the long-term, this knowledge could contribute to the optimisation of treatment protocols leading to improved clinical outcomes in children undergoing neurorehabilitation.

All rehabilitation goals are individual, yet participation [48] and independence in daily life are often mentioned as the most important aims for the rehabilitation of children [49]. The importance of generalisability of motor improvements (i.e. the result of motor learning) and the retention thereof in the daily life of the child is probably not a matter of dispute. Yet, there is a gap between the somewhat constrained reaching movement during an exergame with a robot that is limited to specific movement directions and a goal-directed reaching movement in daily life. Or, in the language of the International Classification of Functioning, Disability and Health [50], practicing ‘body functions’ or ‘capacity’ in a robot might not directly influence ‘performance’ in a daily life environment. While in the robotic condition, the starting position is relatively fixed and the number of movement directions is prescribed and somewhat constrained, reaching in daily life has not such limitations, which leads to many more movement opportunities [51], including numerous movement directions requiring movement control over multiple joints. Furthermore, the use of upper extremities in daily life is mostly a bimanual matter in persons without neurological deficits [52, 53]. While there will much more questions coming up along the way, a randomised controlled trial, as we described it in this protocol, might answer the first question. As there is not enough knowledge available to start with such a study, the conduct of a pilot study is inevitable. Concludingly, this protocol of the planned pilot study is the first step of a series of successive questions and, hopefully, answers, which ultimately leads to an optimised transfer of motor learning improvement into everyday life performance.

### Potential risks and benefits for the participants

It has been stated that scientists need to develop sensitivity for the risks and benefits of the participants when volunteering for a trial [54]. Currently, we do not know whether the blocked or random practice order would be more beneficial. Nevertheless, we assume that the practice schedule described in this study protocol could lead to an improvement in motor functions. Despite that participants will practice only three sessions, according to literature, the resulting 1440 repetitions should induce motor learning as 500 repetitions led to motor learning effects in adults after stroke [37]. Therefore, for all the children participating in this study, a direct benefit can be expected. Especially for such a vulnerable population as children with neurological impairments, the risks of participating in a study should be minimal or, if greater, should be outweighed by the anticipated benefit [55]. Our study provides a safe setting, and we expect no severe adverse events. Potential risks might be fatigue, muscle soreness, a more challenging rehabilitation schedule due to a higher number of sessions than usual and skin irritations due to pressure and friction of the cuffs. The risk-benefit trade-off of this pilot study seems balanced and therewith justifiable.

### Conclusion

After the execution of this pilot study, we will be able to decide upon the questions whether the main randomised controlled trial could be feasible and, if yes, how it should be performed.

## Supplementary information

**Additional file 1:.** SPIRIT 2013 Checklist: Recommended items to address in a clinical trial protocol and related documents

## Data Availability

Not applicable.

## Abbreviations

ANOVA: Analysis of variance
ChARMin: Children’s Arm Rehabilitation Mechatronic Interface
CI: Contextual interference
MACS: Manual Ability Classification System
MA2: Melbourne Assessment 2
MA2_fluency_: Fluency subscale of the Melbourne Assessment 2
nP_norm_: Number of peaks of speed, normalised to the path of the movement
SPIRIT: Standard Protocol Items: Recommendations for Interventional Trials
TIDieR: Template for Intervention Description and Replication
TONI-4: Test of Nonverbal Intelligence, Fourth edition
